# Injectable Hydrogels: From Laboratory to Industrialization

**DOI:** 10.3390/polym13040650

**Published:** 2021-02-22

**Authors:** Jose Maria Alonso, Jon Andrade del Olmo, Raul Perez Gonzalez, Virginia Saez-Martinez

**Affiliations:** I+Med. S. Coop., Parque Tecnológico de Alava. Albert Einstein 15, Nave 15, 01510 Vitoria-Gasteiz, Spain; jandrade@imasmed.com (J.A.d.O.); rperez@imasmed.com (R.P.G.); vsaez@imasmed.com (V.S.-M.)

**Keywords:** injectable, hydrogels, industrialization, scale-up, polysaccharides, regulation

## Abstract

The transfer of some innovative technologies from the laboratory to industrial scale is many times not taken into account in the design and development of some functional materials such as hydrogels to be applied in the biomedical field. There is a lack of knowledge in the scientific field where many aspects of scaling to an industrial process are ignored, and products cannot reach the market. Injectable hydrogels are a good example that we have used in our research to show the different steps needed to follow to get a product in the market based on them. From synthesis and process validation to characterization techniques used and assays performed to ensure the safety and efficacy of the product, following regulation, several well-defined protocols must be adopted. Therefore, this paper summarized all these aspects due to the lack of knowledge that exists about the industrialization of injectable products with the great importance that it entails, and it is intended to serve as a guide on this area to non-initiated scientists. More concretely, in this work, the characteristics and requirements for the development of injectable hydrogels from the laboratory to industrial scale is presented in terms of (i) synthesis techniques employed to obtain injectable hydrogels with tunable desired properties, (ii) the most common characterization techniques to characterize hydrogels, and (iii) the necessary safety and efficacy assays and protocols to industrialize and commercialize injectable hydrogels from the regulatory point of view. Finally, this review also mentioned and explained a real example of the development of a natural hyaluronic acid hydrogel that reached the market as an injectable product.

## Objectives and List of Content

The goal of this review article is to discuss the open issues concerning the up-scaling and industrialization of the fabrication of injectable hydrogels. Here, we include the list of content of the manuscript so that the reader may easily understand the subject matter of it.

*1—Introduction*

*2—Injectable Hydrogels: Properties and Synthesis Techniques*

*3—Characterization Techniques*

*3.1—Physicochemical Characterization*

*3.2—Structural/Morphological Characterization*

*3.3—Thermal and Mechanical Characterization*

*3.4—Biological Characterization*

*4—Process from the Hydrogel Obtention in the Lab to its Industrial Production*

*4.1—Scale-Up Key Technical Parameters: Design and Development*

*4.2—In-House example: Fabrication of an Injectable Hydrogel*

*4.3—Regulatory Aspects*

*5—Conclusions*

## 1. Introduction

Hydrogels are three-dimensional physically or chemically crosslinked polymeric networks from natural or synthetic origin, with an intrinsic hydrophilic character due to their functional groups. They have unique properties, such as high water content, softness, flexibility, porosity, permeability, and biocompatibility and a very high affinity for water and other body fluids. These properties resemble those of many soft living tissues, which opens up many opportunities in biomedical and pharmaceutical applications [1,2]. In this regard, drug delivery systems based on hydrogels have been developed in order to provide drug reservoirs for treating conditions of soft tissues such as for example bladder diseases [3]. Similarly hydrogels have been synthetized as scaffolds for cartilage tissue engineering [4]. Therefore, these properties make hydrogels an ideal candidate to provide a better adaptation after the implantation.

**Figure 1:**
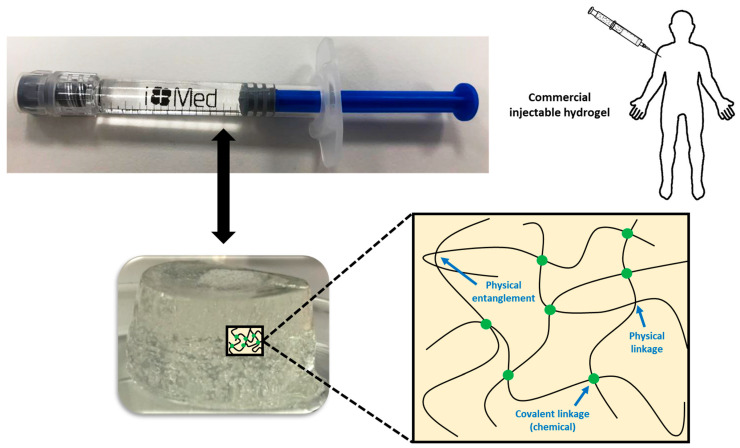
Physical interactions and chemical linkages of the chemical structure of an injectable hydrogel.

In some cases, chemical and physical networks might coexist in one hydrogel (Figure 1). Physical hydrogels are formed by reversible physics interactions, and they can be dissolved by changing environmental conditions [5]. Otherwise, chemical hydrogels are mainly formed by the covalent bonds that appear after certain chemical reactions, and they are recognized as stable or permanent in physiological conditions. These hydrogels can be prepared by using a hydrophilic monomer polymerized in the presence of a polyfunctional crosslinking agent [6] or by the direct crosslinking of water-soluble monomers in the presence of a free-radical generating initiator that can be activated by radiation (light, heat, etc.) or by chemical reactions (redox). However, some of these pathways often result in materials containing significant levels of residual unreacted monomers that are often toxic and could lixiviate out from the hydrogel. Thus, a purification step is needed, and this can take up from several days to weeks to be completed. The selection of non-toxic oligomers or macromonomers (e.g., polyethylene glycol acrylate derivatives) could be an alternative [7].

It is also possible to avoid the need for purification by using water-soluble polymers such as many polysaccharides. However, sometimes, they also need some kind of crosslinking agent, and the need to purify unreacted molecules of it comes back again. Among them, the most common glycosaminoglycan found in many living tissues is hyaluronic acid (HA) or hyaluronan. HA is a linear glycosaminoglycan made up of repeating units of N-acetyl-d-glucosamine and d-glucuronic acid with the monosaccharide units linked together via alternating -1,3 and -1,4 glycosidic bonds [8]. In physiological conditions, HA takes the form of a sodium salt that is negatively charged and highly hydrophilic [9]. HA can be crosslinked by several of the methods mentioned before to form hydrogels for several biomedical applications. However, the implantation of pre-formed hydrogels at a desired site in the body demands an invasive surgical procedure that can cause the patient’s pain and discomfort as well as the cost and time [10,11]. For this reason, injectable hydrogels are becoming more interesting for many of these applications.

As hydrogels are becoming more useful materials in many therapeutical approaches, the challenge has arisen for research laboratories and the biomedical industry to get an effective technology transfer and scale-up of processes. For this reason, many aspects that affect the scaling to an industrial process should be considered right from the beginning of any new development. This industry-focused point of view is ignored by scientists most of the time, and that is one of the reasons why many good ideas and potential products never reach the market, and thus, the patient, who would never benefit from them. While the exploration on synthesis is indispensable, the construction of new formulations using common materials, simple methods, and facile design must become a preferential choice, without forgetting to assess the influence of the process procedure on the properties of the fabricated hydrogel. Not just the chemical, physical, and biological parameters must be controlled in order to guarantee a successful process, but also economic, legal, and regulatory aspects. The product obtained in the laboratory should have not only the same characteristics and performance as that at the larger scale but also must be obtained by reproducible and controllable processes. From the point of view of regulation, the biomedical field is very strict in parameters such as sterility and toxicity.

For example, in the characterization of injectable hydrogels, several parameters must be determined, since they are necessary for their industrialization. These requirements are listed in European Pharmacopoeia (Ph. Eur.) and should follow International Organization for Standardization (ISO) norms or American Society for Testing Materials (ASTM) standards. Therefore, they must possess a list of specifications (characteristics of the commercial product) with a description of validated analytical techniques used for the measurement of these specifications. Thus, to get the CE marking and the consequent commercialization of the product, the list of specifications of the injectable product must conform the established and tolerable limits for its future application.

This paper summarizes all these aspects and shows a real example of a development of a natural crosslinked hydrogel based on hyaluronic acid that reached the market in the form of an injectable product.

## 2. Injectable Hydrogels: Properties and Synthesis Techniques

Injectable hydrogels have the right physicochemical properties to be injected in situ into the body and therefore, they have attracted significant interest in drug delivery, tissue engineering, and as dermal fillers [12,13,14,15]. Thus, one of the most important factors to be considered is the viscosity of the polymer solution, as this feature is advantageous in minimally invasive surgical procedures. Some injected hydrogels trigger a wide variety of inflammatory, immune-mediated, local, or systemic adverse reactions that appear early or late, illustrating that biocompatibility and non-toxicity are important criteria of a good injectable hydrogel system. Another important factor is the hydrogel porosity, where highly inter-connected networks are preferred, as they facilitate better movement of nutrients and adaptation to surrounding tissues. This also relates to the proper mechanical properties (tensile strength and modulus, compressive stress and modulus, shear stress, stiffness, storage and loss moduli, fragility, mesh size, and density, among others) [16], as the hydrogel should withstand the repetitive deformation that occurs in the mechanically dynamic environment in the body.

An injectable hydrogel is generally based on the idea that some biomaterials can be injected as liquid into human body, and then, an in situ solid hydrogel is formed [17]. However, injectable hydrogels are not only all those that gel once they have been injected into the human body. Indeed, hydrogels with shear-tinning and self-healing properties are also recognized as injectable biomaterials, since they can also be injected directly into the gel state [18,19,20]. All in all, these injectable biomaterials require a better control of gelation kinetics on the process procedure, which requires transporting the sol or the pre-gel to a targeting site through an injection device. They should fill the void space and especially have the desired stability at the injected site. This means that the sol–gel transition of an injectable hydrogel should happen within a restricted time interval to get the right injectability. Moreover, the injection procedure for injectable hydrogels could also influence the structure and the properties of the harvested bulk gel, reaching a poorer performance against deformation than the corresponding in situ formed gels. However, as in other hydrogels, the mechanical properties and durability can be tuned by varying the portion of the monomers or oligomers, the molecular weights, and the crosslinking density of the hydrogel.

The typical crosslinking strategies applicable for the synthesis of ordinary hydrogels are also applied for the development of injectable hydrogels. So far, injectable hydrogels can be crosslinked by different synthesis mechanisms, and in their chemical structure, physical and chemical linkages can be found and coexist between them [21,22]. Physical linkages include electrostatic interactions such as electrostatic forces, ionic and hydrogen bonds, van der Waals forces, π-interactions, or hydrophobic interactions. Hydrogels that only or mainly possess physical interactions in their structure can be merely prepared without any additional reactive reagents, but they are slightly mechanically weak to preserve themselves from the ambient influences such as pHs and temperatures for a long time, when injected into the body. On the other hand, in injectable hydrogels in which chemical linkages are majority and dominant forces Diels–Alder reactions, Michael-type additions, Schiff base reactions, enzyme-mediations, thiol exchange/disulfide crosslinking, and click chemistry can be included. These hydrogels can be triggered by external stimuli (temperature, pH, light, electric/magnetic fields, ultrasound, and enzymes), and they have been shown to possess relatively higher mechanical stability and physicochemical properties with greater durability over time due to the formed strong covalent bonds.

In general, HA-based injectable hydrogels are prepared using chemical crosslinking agents such as 1,4-butanediol diglycidyl ether (BDDE) and divinyl sulfone (DVS) to overcome the very short half-life of HA, several hours in the body, due to its fast enzymatic degradation by hyaluronidase [23]. However, despite the use of chemical crosslinking agents, the stability and longevity of these hydrogels usually shows a persistence of only 6 months in vivo, and they require repeated injection to maintain their efficacy [24,25]. Therefore, there is an unmet need to develop new injectable materials that are safer and longer lasting.

## 3. Characterization Techniques

The wide range of synthetic procedures to obtain injectable hydrogels can allow scientists to endow these biomaterials with desirable physical, chemical, and biological properties. For that reason, in order to obtain injectable hydrogels with the desired tunable properties and fulfill the necessary requirements and specifications for its future application, the characterization of hydrogels is of special interest. Over the last decades, intense and deep studies have been carried out in the field of injectable hydrogels, and for this reason, nowadays, a wide variety of characterization techniques can be found. These characterization techniques can be divided in the following: physicochemical, structural/morphological, thermal, mechanical, and biological characterization.

### 3.1. Physicochemical Characterization

Physicochemical properties of crosslinked hydrogels polymeric network provide chemical and physical characterization in terms of the different functional groups and their intermolecular interactions. In fact, these different functional groups play an important role in the desired crosslinking mechanism and therefore, in hydrogels’ final properties. The following combination of various characterization methods can be used to identify injectable hydrogels’ performance and quality that it must be within the acceptable conformities range previously defined by standards.

#### 3.1.1. Gelation Time

**Figure 2:**
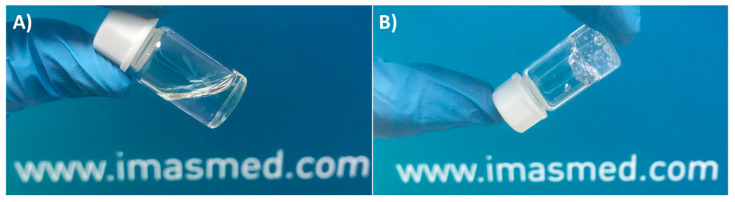
Example of a gelation time measurement by inverted vial method.

Although the study of gelation time and kinetics for the injection of already synthetized hydrogels into the human body has no sense, it is of special interest, especially in the preparation of hydrogels for in situ applications and subsequent crosslinking into the human body once injected [26]. The study of gelation is an easy, simple, and reliable technique that is generally governed by the mechanism responsible for the gel formation (physical or chemical interactions). Gelation can be determined by different methods, but, nowadays, the most used techniques are the “tabletop”-called ones, as is the case of the test vial inverting method (Figure 2), which possesses a rheological basis [27].

The elastic rheological response of a gel sample is used for the qualitative diagnosis “tabletop” experiments without using a rheometer. An inverted vial test is particularly useful for evaluating the limit in which the sample starts to have a gel-like behavior, and it is based on visual observation and feel, noting if the sample flows under its own weight. In fact, gel-like samples will not flow, whereas a viscous but inelastic sample (viscous liquid) will show a measurable flow.

This experiment must be performed in a bath at constant temperature and the physical state of tested samples is noted (viscous liquid or gel) by turning a test tube or vial containing the sample. Then, the bath temperature is changed and again, the new physical state of tested samples is registered. The test is repeated as many times as you want in the temperature range of interest. Despite its simplicity, nowadays, inverted tube tests are used to study the injection of hydrogels into the human body for in situ biomedical applications at temperatures below and above human body temperatures (simulated physiological conditions, 37 °C) [28,29].

#### 3.1.2. Rheology

Rheology can be used to determine the rheological properties of injectable hydrogels by establishing a relationship between deformation or flow and applied stress [30,31]. Rheology is an ideal method for viscoelastic properties determination, as it is sensitive, quick, requires small hydrogel amount per measure (≈1 g), and additionally, it can provide information about hydrogels crosslinking grade, molecular weight, and structural homogeneities/heterogeneities, among others. These rheological properties are the most relevant features of injectable hydrogels since stability, usability, and application in the biomedical field is determined by them.

Viscosity (η), elastic modulus (G′), viscous modulus (G″), complex modulus (G*), and loss factor (tan δ) are the primary rheologic parameters used to characterize injectable hydrogels. However, there are other relevant parameters that could apport valuable information about the rheological properties of the hydrogels (Table 1) [32]. A combination of the viscoelastic properties of Table 1 can be measured in all injectable hydrogels, but a characteristic much higher G’ modulus than G″ modulus is always observed independently of the applied frequency (G′ > G″). This fact is an unmistakable sign that the crosslinking of the polymeric networks and the resulting hydrogel formation have been carried out in a satisfactory manner. Hence, G´ is the most determinant and appropriate parameter to differentiate and compare injectable hydrogels since diverse factors (e.g., crosslinking degree, chain entanglements, concentrations, and molecular weight) have a direct effect in hydrogels strength and therefore, in G′ value [33,34].

**Figure 3:**
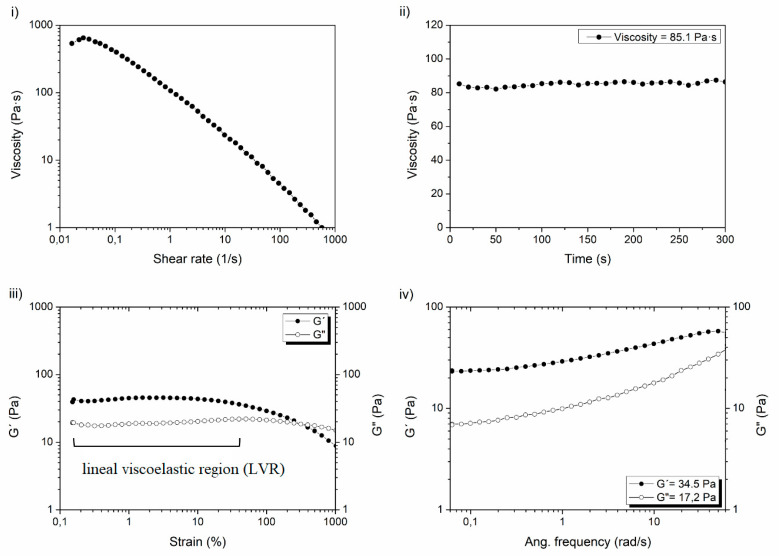
Steps of proposed protocol for standardization of the rheological characterization of injectable hydrogels: (**i**) determination of viscosity vs shear rate; (**ii**) viscosity vs time; (**iii**) elastic modulus (G′) and viscous modulus (G″) vs strain; (**iv**) elastic modulus (G′) vs Ang. frecuency.

There exist several rheological protocols for the study of the viscoelastic properties, but the lack of similarities between them derived from different procedures makes it difficult to compare the rheological properties of different hydrogels [35]. For this reason, at our labs at i+Med, the following protocol is applied to standardize the rheological characterization of injectable hydrogels, measuring the parameters that could offer the most rewarding information about the hydrogels: firstly, viscosity measurements and then, the elastic (G′) and viscous (G″) modulus determination. An example of this proposed protocol with its corresponding steps can be seen in Figure 3.

i. Determine the viscosity of hydrogels as a function of shear rate by a flux shear rate sweep. In this case, a shear thinning behavior of the hydrogels must be observed to confirm the injectability of the hydrogels, unlike non-crosslinked HA solutions that show a Newtonian behavior.
ii. Calculate viscosity values as a function of time at constant shear rate (e.g., 1 s^−1^) by a flux time sweep. In this case, viscosity values must maintain almost constant over the time without fluctuations to accept the measure.
iii. Before G′ and G″ modulus determination, an oscillatory strain sweep test must be performed in order to know the linear viscoelastic region (LVR) in which concrete strain must be selected and fixed for the subsequent oscillatory frequency sweeps.
iv. Finally, after the previous assessment and the appropriate selection fixing a certain strain (e.g., 1%), elastic (G′) and viscous (G″) modulus can be measured correctly by oscillatory frequency sweeps.

#### 3.1.3. Syringeability and Injectability Evaluation

Syringeability and injectability are important parameters to take into account in the field of injectable hydrogels, which are very closely and directly related to rheological properties. Syringeability is related to the facility to release the hydrogel through a needle, and injectability, on the contrary, evaluates the syringe performance on a subcutaneous injection by measuring the force required to perform the administration via a syringe.

Nowadays, there are not any international standards or methods to regulate how to perform these tests, but in the literature, several syringeability and injectability tests can be found [29,36,37]. However, among them, the experimental procedure of Moreira et al. [38] could be the most useful due to its facility and simplicity. In the determination of both parameters, the gauge of the needle must be the same and conforms to the one used for the subcutaneous injection of hydrogels in the industrial field, in which gauges range from 18–21 G to 26–27 G.

On the one hand, syringeability is measured as the percentage of hydrogel effectively expelled from a syringe. For that, a constant force (e.g., 50 N) during a certain time (e.g., 5 s) is applied to a syringe and quantitatively studied in terms of the residual hydrogel mass retained in the syringe (Equation (1)).

(1) $$Syringeability\;\left(\%\right)=\frac{mass\;expelled\;fron\;the\;syringe}{mass\;of\;the\;sample\;before\;injection}\cdot 100$$

On the contrary, injectability is determined in terms of the force required for injection of the hydrogel in a compression test using a universal machine monitoring force versus displacement under a constant crosshead speed. In this case, initial glide force and then, the maximum force detected during the experiment are quantified, and in both cases, their values must be below 30 N, which is the limit force for an acceptable subcutaneous injection.

#### 3.1.4. Spectroscopy and Spectrometry Techniques

The presence of different functional groups in synthetized hydrogels has an important effect in the physicochemical properties, such as rheological properties, degradation, and swelling ability, among others. Therefore, the more and better the chemical composition of the polymeric network is known, the more capacity it will have to be able to adjust and regulate the polymeric characteristics and thus finally obtain the desired final properties of synthetized injectable biomaterial.

Solution- and solid-state nuclear magnetic resonance spectroscopy (NMR) [39,40], Fourier-transform infrared spectroscopy (FTIR) [41,42], ultraviolet-visible absorption spectroscopy (UV-Vis) [43], and Raman [44] spectroscopy are the numerous spectroscopy techniques that have been widely employed to determine the structure of the injectable hydrogels. These spectroscopy techniques are based on the interaction between radiated energy and matter, and essentially, they can provide us with qualitative results related with the polymeric structure and composition of the polymer; that is, they do not create quantitative results on their own. More concretely, they can be employed to verify the crosslinking reaction of synthetized hydrogels by the appearance or disappearance of some signals, analyze specific molecular structures and physicochemical interactions after crosslinking reactions to corroborate hydrogels synthesis (e.g., formation of H-bonds or π–π interactions), identify the creation or destruction of chemical links by the increase or decrease in the intensities of specific bands or signals, and so on. However, although they do not give us directly quantitative results, their results can be used to calculate some quantitative parameters, such as the degree of crosslinking by the degree of modification (MoD) or crosslinking ratio (CrR) parameters, which can be obtained from NMR spectroscopy integrating the signals of the spectra [45].

On the other hand, spectrometry techniques are able to get quantifiable results since they are based on the application of spectroscopies. This is the case of mass spectrometry, which is one of the major analytical techniques used to examine mass, elemental composition, and chemical structure of a molecule. It possesses high precision and accuracy for molecular weight determination, high detection sensitivity, and it usually is coupled to electrospray ionization (ESI), inductively coupled plasma (ICP), and matrix-assisted laser desorption/ionization (MALDI). This last is of special interest, since this technique is helpful to achieve high resolution for synthetic and biological macromolecules as well as polymers [46].

#### 3.1.5. Swelling Ability

**Figure 4:**
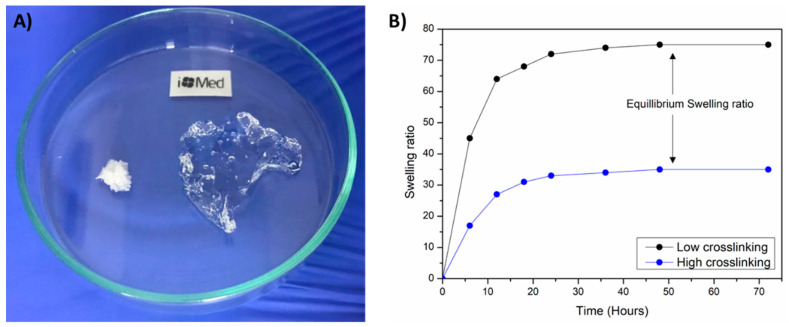
(**A**) Swelling ability of hydrogels from lyophilized state and (**B**) equilibrium swelling ratio determination.

The swelling capacity of injectable hydrogels is of utmost importance since thanks to this property, drugs or other agents can be loaded into hydrogels to provide controlled drug release properties, and thus, supply an added value to these biomaterials for biomedical applications (Figure 4) [47,48,49]. There are a lot of methods available for swelling capacity determination but i+Med proposed the following one. The swelling ratio of lyophilized hydrogels were measured by the immersion of a known weight of samples in a medium which simulates human physiological conditions (phosphate buffer saline (PBS) at 37 °C) for 48 h. When the equilibrium swelling is reached, the swollen hydrogels are weighted after the excess of water was removed with a filter paper. The use of an analytical balance with high sensitivity (at least 10^−3^–10^−4^ g) is recommended to weight the swollen hydrogels. Finally, the equilibrium swelling ratio was calculated using the following equation (Equation (2)):

(2) $$Equilibrium\;Swelling\;Ratio\;=\frac{W_{s}-W_{d}}{W_{d}}$$

where *W_s_* and *W_d_* are the hydrogels weight at equilibrium swelling state and at dry state, respectively.

Figure 4A shows the lyophilized hydrogel in its initial state and to its right, the hydrogel in its equilibrium swelling state after immersing it in PBS. Moreover, Figure 4B shows the swelling ratio of an injectable hydrogels over the time after immersion of the lyophilized sample in PBS. Once the swelling ratio is constant over time, the equilibrium swelling ratio can be determined. It is worth highlighting that injectable hydrogels with a higher crosslinking degree generally possess lower swelling ability due to the higher quantity of linkages of the polymeric network that result in a lower elasticity and mobility of the polymeric chains, with the consequent lower capacity to absorb PBS.

#### 3.1.6. Stability and Degradation

The stability of a hydrogel refers to maintaining the same properties for a period of time under certain conditions. This phenomenon is affected by numerous factors as solvents, temperature, moisture, pH, exposure to radiation, enzymatic degradation, and so on [50]. For this reason, it is necessary to study the stability and degradation of hydrogels, simulating as well as possible the medium in which the hydrogels are pretended to be injected in the future application.

An easy, simple, and reproducible in vitro method for the measurement of injectable hydrogels stability in the laboratory is to monitor their degradation with respect to the weight loss of initially weighted hydrogels (*W*_0_) as a function of incubation time. Then, at some specific times, hydrogels are removed from PBS and weighted (*W_t_*). The remaining hydrogel mass ratio was calculated according to Equation (3):

(3) $$Remaining\;Mass\;\left(\%\right)=\;100-\left(\frac{W_{0\;}-W_{t}}{W_{0}}\;\cdot 100\right)$$

In these assays, as well as in swelling ratio measurements, a hydrolytic medium that simulates human physiological conditions (phosphate buffer saline (PBS) at 37 °C) is employed, and to reach an enzymatic medium, enzymes that degrade the polymeric chains of the hydrogel is used. For example, hyaluronidase and lysozyme enzymes are the most used enzymes in the stability tests of hyaluronic acid- and chitosan-based hydrogels [51]. However, these hydrogels can have a very high stability at 37 °C and take months to years to degrade. Therefore, there are also accelerated stability studies to have stability results of the hydrogels in the shortest possible time [52]. For instance, in these accelerated studies, the test conditions in the adapted stability chamber are changed to 40 °C and the relative humidity is increased to 75%.

Moreover, whether it is of interest that the hydrogel degrades rapidly (for instance, in tissue engineering applications), or if you want hydrogels to have high stability so that it lasts in the human body as long as possible (dermal fillers), it is necessary to control the degradation products that are created during the process. In fact, cytotoxic degradation subproducts can be created, but to overcome this issue, injectable hydrogels based on biopolymers with less or no cytotoxic degradation products are being employed in the last decades, such as hyaluronic acid, alginate, cellulose, starch, silk, and chitosan, among others [50].

#### 3.1.7. Other Physicochemical Characterization Techniques

In the characterization of injectable hydrogels, there are other physicochemical characterization techniques that are less common and less used, but which may be of great importance in determining certain properties. Among these techniques can be found dynamic light scattering (DLS), zeta potential, and diffraction techniques.

Dynamic light scattering (DLS) is a technique from which lots of parameters and properties from the polymeric systems can be obtained, such as molecular distribution, particle size distribution, molecular weight of the polymer, molecular weight distribution, polydispersity index, hydrodynamic size, shape, structure, aggregation state, and biomolecule conformation, among others [53]. Moreover, it possesses several advantages as it is noninvasive, has high accuracy and reproducible measurements, short experiment times (minutes), and low apparatus costs. However, DLS has a limited utilization for heterogenous and non-spherical polymeric particles as well as when certain amounts of aggregates are presented.

Moreover, the zeta potential of a hydrogel can be measured in ionic solution when they are charged and therefore, the electric potential appeared in the polymeric material. A zeta potential close to 30 mV is usually chosen to get stable particles. Thus, an absolute value higher than 30 mV means a stable measurement, while zeta potential values lower than 30 mV are related to particle aggregation and therefore, with instable measurements [54]. Additionally, as this technique is very sensitive to environmental changes (pH and ionic strength), it is difficult to provide precise and repeatable zeta potential measurements.

Diffraction techniques such as small-angle X-ray scattering (SAXS) and X-ray diffraction (XRD) are employed to get the molecular order of a system and nanoscale structures in crystalline materials at atomic scale. Although this technique has a limited application in disordered materials with few crystalline zones, as is the case of hydrogels, there are some few cases described in the literature in which diffraction techniques help determine the formation of inclusion complexes and the resulting gel formation [55]. For example, Li et al. [56] used XRD to confirm the formation of a hydrogel basing on the pattern of the crystalline structure of chitosan polysaccharide. Moreover, XRD can also be useful to study the effect of crosslinking agent on the hydrogels morphology [57] and to identify nanoparticles in hydrogel systems [58]. Finally, an example of the utility of SAXS in hydrogels would be the study of Waters et al. [59], in which the polymerization process of developed hydrogel was controlled and monitored by this technique.

### 3.2. Structural/Morphological Characterization

Structural or morphological characterization is one of the most used techniques in the characterization of injectable hydrogels, in which the porous microstructure of the hydrogel can be observed. This microstructure is directly related with the swelling ability of the hydrogels, since the water is entrapped in these regions. Scanning electron microscopy (SEM), transmission electron microscopy (TEM), atomic force microscopy (AFM), scanning tunneling microscopy (STM), and confocal microscopy are the techniques used to study the morphological characterization on hydrogels.

**Figure 5:**
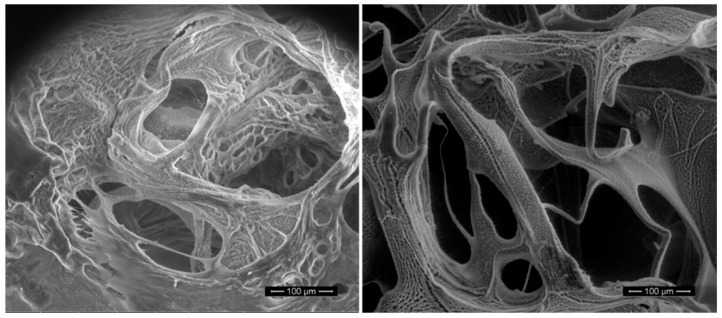
Cross-section SEM images of freeze-dried injectable hyaluronic acid hydrogels.

SEM and TEM techniques provide the three-dimensional image of the structure, topography, composition, pore size, pore size distribution, and aggregations and dispersions of polymeric network, among others [60]. The main difference between them is the magnification at which microscopes can work. While SEM can be controlled up to the range of six orders of magnitude about 10 to 500,000 times to observe microscale structures (Figure 5), the higher special resolution down to the level of atomic dimensions (<1 nm) obtained with TEM leads to the analysis of materials at nanoscale. The main disadvantage of both techniques is the necessity of drying the samples by the lyophilization process before analysis and therefore, the difficulty to measure nonconductive specimens. This problem of getting imaging faults due to the insufficient deflect of the electron beam can be solved by coating an ultrathin layer of an electrically conductive material, such as a gold layer. However, nowadays, there are variants of both techniques that permit the imaging of the hydrogels in their natural state without magnification or preparation, as is the case of environmental SEM (eSEM) and wet scanning TEM (STEM) [61,62]. While eSEM works at high humidity environment and images can be obtained under partial water vapor pressure, STEM enables the observations of totally emerged samples in liquid state, even though there are several micrometers of water. Another characterization technique that varies from SEM and enables the structural characterization of high-water content materials as hydrogels is cryo-scanning electron microscopy (cryo-SEM) [63]. Cryo-SEM has the potential to exhibit the most authentic insights in preserving the structure of hydrated biological specimens, such as polysaccharide hydrogels, while the solvent or dissolved components are maintained. It is based on the cryo-fixation of water by forming ice-crystals and vitrification process is involved [64]. However, in order to obtain high-quality cryo-SEM results, the cryo-fixation process must be controlled perfectly, since poor cryo-fixation process control could lead to structural damages of the polymeric network due to the formation of ice-crystal aggregates that would alter the hydrogel structure.

AFM, SEM and confocal microscopy are other less used high-resolution techniques to characterize the morphology of hydrogels. Even so, AFM can provide information about the surface properties of the nonconductive materials by creating a topographic image of the hydrogel surface, and SEM can give the conformation of a gel by imaging biomolecules in their native conditions without preparation, and finally, confocal microscopy gives improved high-quality images by acquiring point-by-point images and allowing 3D reconstruction of complex fluorescent morphologies [65,66].

### 3.3. Thermal and Mechanical Characterization

Other types of characterization techniques are thermal and mechanical analyses of injectable hydrogels, which are used every day in the laboratory and at the industry scale neither for the research or development of new materials nor quality control or assurance tests. On the one hand, thermal analyses are based on the changes in the structure that hydrogels suffer when temperature is changed; on the other hand, mechanical tests give information about the strength and stiffness of crosslinked polymeric chains of the hydrogels when a loading is applied. Thermal analysis techniques provide an insight into specific thermal properties of the hydrogel products, such as thermal transitions, decomposition temperatures, specific heat, glass transition temperatures, thermal stability, and degree of crystallinity. Meanwhile, Young´s modulus is the most measured parameter to determine the mechanical properties of injectable hydrogels. Among these techniques, thermogravimetric analysis (TGA), differential scanning calorimetry (DSC), dynamic-mechanical thermal analysis (DMTA), and compressive stress/strain studies can be found.

In TGA measurements, the mass of the tested hydrogel is monitored over the time as temperature changes, and it could give physical (phase transition, adsorption, desorption and absorbed water content) or chemical information (chemisorption, thermal decomposition, thermal stability, and oxidation or reduction reactions). Moreover, in thermogravimetric curves with several mass loss stages, the percentage of mass loss for each step can be measured and associated with the degradation of each component of the injectable hydrogel [67].

The DSC thermo-analytical technique is a very useful and sensitive technique to quantify the heat required to increase the temperature of a sample comparing to a reference as a function of temperature. In this case, the temperature at which transitions of the polymer took place through changes in heat capacity can be measured, which lead to the study of glass transition temperature (T_g_), crystal structure, degree of crystallinity, crystal size distribution, and water content [68].

DMTA allows the quantification of the mechanical properties of hydrogels under oscillatory force and as a function of temperature, time, frequency, and strain [69]. DMTA provides the viscoelastic properties of a sample by representing storage modulus (E′), loss modulus (E″) and tanδ (loss factor) versus temperature. This technique is of special interest in industries for the development of hydrogels and quality controls. It usually works at temperature ranges from −180 to 600 °C, and it is helpful especially for the identification of transition regions, such as T_g_ determination.

Finally, the mechanical features of hydrogels can be measured by compressive stress/strain studies and getting stress (kPa)–strain (%) curves. From this curves, tensile and ultimate strength, elongation at break, and Young´s modulus applying linear regression can be measured [70]. It must be pointed out that in mechanical tests, preparation of the sample is very important, the since the samples to be tested must have always the same geometry (length × width × thickness) so that the obtained results can be comparable between them.

### 3.4. Biological Characterization

In general, injectable hydrogels demonstrated good biological response and biocompatibility, since they are usually synthetized from biopolymers obtained from the natural resources, as is the case of hyaluronic acid, chitosan, alginate, and heparin, among others [71]. That is why scientists are developing every day more and more biocompatible injectable biomaterials to be employed and applied in the different areas of the biomedical sector, such as controlled drug or gene delivery, wound-healing, tissue engineering, and biosensing [72].

At this point, it is necessary to differentiate between the biological characterization techniques that on the one hand give us information and help demonstrate the efficacy and application of the injectable hydrogels in previously mentioned biomedical areas, and on the other hand, protect human life from dangerous and hazardous biological responses derived from the use of these injectable materials. In the first group, examples of in vitro biological characterization techniques are cell proliferation and differentiation [73], wound-healing assays [74], and antimicrobial or anti-inflammatory tests derived from the sustained release of drugs from hydrogels [75]. Once the efficacy of the injectable product is demonstrated in vitro, in vivo performances and animal testing can be carried out for the final demonstration efficacy of the biomaterial.

On the other hand, it is first necessary to test the safety of the developed injectable hydrogels by a biological testing and safety assessment. For that, a preclinical study, in which the injectable product safety is demonstrated, must be performed and submitted to the corresponding notified body. A notified body is an entity authorized by the corresponding government agency or authority to assess and certify the conformity of a medical device with the requirements of the EU legislation governing medical devices and with applicable harmonized standards. Nowadays, injectable hydrogels used as implants, such as dermal fillers, are recognized as medical devices. The guidance in which the necessary and minimum biocompatibility tests to ensure the safety of medical devices that must be performed in the preclinical study are included in the UNE-EN-ISO 10993:2018 standard [76].

## 4. Process from the Hydrogel Obtention in the Lab to Its Industrial Production

The process for the production of injectable hydrogels should be planned, starting from the quality by design approach (QbD). QbD pre-defines the properties of the targeted injectable hydrogel. It is based on statistical, analytical, and risk-management methods applied to understand the product and its fabrication process [77].

The purpose of a QdB is to assess how the formulation of the injectable hydrogel and the fabrication parameters influence the characteristics of the formulation. QdB involves the definition of a quality target product profile (QTTP) and the critical quality attributes (CQAs) of the formulation [78]. QTTP refers to parameters of the product such as indication, route of administration, dosage form, packaging, stability, dispersibility and moisture, while CQAs relates to components, contents uniformity, water content, microbial content, and physicochemical properties (pH, osmolality). QTTP and CQAs can be established according to recommendations from the European Medicines Agency (EMA): ICH guideline Q8 (R2) on pharmaceutical development. Available from: https://www.ema.europa.eu/en/documents/scientific-guideline/international-conference-harmonisation-technical-requirements-registration-pharmaceuticals-human-use_en-11.pdf, and US Food and Drug Administration (FDA): Guidance for industry: nasal spray and inhalation solution, suspension, and spray drug products—chemistry, manufacturing, and controls documentation. 2002. Available from: https://www.fda.gov/media/70857/download.

**Figure 6:**
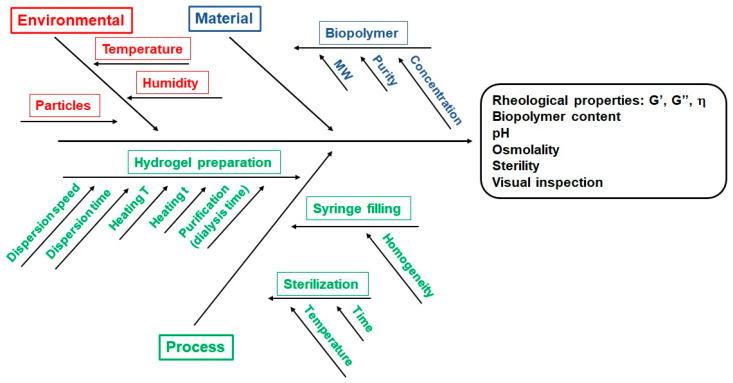
Ishikawa diagram for the fabrication of the injectable hydrogels.

After the CQAs of the final product are established, the next step is to define a risk analysis of the effect of different parameters in the CQAs of the formulation: ICH guideline Q8 (R2) on pharmaceutical development. Available from: https://www.ema.europa.eu/en/documents/scientific-guideline/international-conference-harmonisation-technical-requirements-registration-pharmaceuticals-human-use_en-11.pdf. Such parameters connect with the features of the starting materials (biopolymers, active ingredients, solvents, ingredient ratios) and with the stages in the fabrication process (i.e., heating temperatures, reaction time, agitation speeds). Such a relation can be outlined through an Ishikawa diagram, which clarifies the parameters that might modify the CQAs of the formulation (Figure 6).

### 4.1. Scale-Up Key Technical Parameters: Design and Development

#### 4.1.1. Rheological Parameters

Injectability of the hydrogels relies on their capacity to pass through a syringe needle; however, this parameter does not guarantee itself the performance of an injectable gel. Therefore, the characterization of the precursor solution is compulsory not only for the scaling up of the fabrication process but also for providing the medical professionals the most accurate information about the actual behaviour of the hydrogel.

The most important rheological parameters from a practical point of view are (a) shear response, i.e., easiness for injection; (b) recovery time (time for placement); and (c) yield stress, which refers to the retention of the hydrogel solution at the defect point [79].

For thermosensitive hydrogels, the rheological properties should be recorded vs. temperature, and the time needed for gelation at 37 °C should be characterized. Artificial intelligence provides an insightful tool for predicting rheological behaviors, reducing the time and cost of analysis [80].

In scale-up processes, the rheological fluid behavior must be normalized in order to foresee the flow behaviors by comparison with the common standardization function (master curve) and to predict non-Newtonian fluid parameters based on Newtonian models [81].

In that regard, for Newtonian fluids, shear stress is related to the shear rate by the dynamic viscosity, which depends on the hydrogel and the temperature, but for non-Newtonian fluids, the shear stress may also depend on time [82]. Viscoelastic hydrogels are an example of it, since they recover the initial conformation a while after a deforming force was applied. The Herschel–Bulkley equation simplifies complex rheological properties and their interactions [81] and is usually employed for non-Newtonian fluids in order to compare rheological properties.

#### 4.1.2. Process Parameters

##### Dispersing Machinery

**Figure 7:**
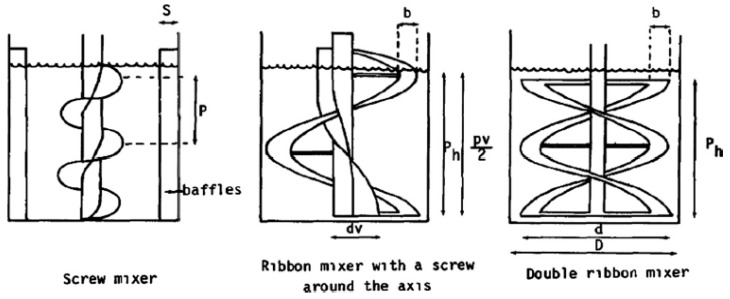
Ribbon mixer with a screw around the axis, screw mixer with four baffles, and double ribbon mixer propellers. Reproduced with permission from reference [83].

Usually, natural hydrogels are produced in a short time by mixing a solid powder phase (hydrogel precursor). The mixing process must ensure that a homogenous liquid phase is formed into the reactor. For that, different propeller configurations depending on the solution viscosity can be applied. A ribbon mixer with a screw around the axis, screw mixer with four baffles, and double ribbon mixer propellers were successfully employed for the mixing of Newtonian fluids [83] (Figure 7).

##### Time

In order to establish the mixing time, dimensional analysis is performed based on Reynolds and Archimedes numbers. Moreover, it is also compulsory to consider the speed and the geometry of the stirrer, and the parameters of the fluid, such as dynamic viscosity and density [84].

##### Temperature

The temperature must stay constant during the whole chemical process. In this regard, thermo-dependant processes, for instance volume and viscosity changes, gelation, etc. challenge the process control. Changes in viscosity demand the power input of the stirrer motor to adapt in order to achieve a constant speed during the agitation process [82]. For this purpose, sensors and software are developed to control specific chemical processes [85].

##### Purification of the Hydrogel

Once the chemical modification of the hydrogel occurs, the next step involves the purification of the resulting matrix. This stage may proceed via precipitation followed by washing or just by a washing step. Washing allows for the removal of the non-reacted reagents and, if required, for the pH regulation of the hydrogel matrix. Washing takes place by direct contact of the hydrogel with an aqueous phase or by the use of membranes in a dialysis process. In this regard, the use of dialysis membranes of defined pore size permits the selective elimination of substances from the gel matrix. Currently, there are commercial solutions available for the up-scaling of the dialysis step.

##### Filling of Syringes with Hydrogels

After purification of the hydrogel, usually, a correction in the concentration of hydrogel-producing polymer is required depending on the application of the injectable hydrogel. Subsequently, the hydrogel is filled in syringes, and the resulting product is sterilized. Finally, the filled syringe is labeled, visually inspected, and blister packed. Depending on the manufacturing process, the sterilization stage may occur as a final step of the fabrication work-flow. The rheological parameters of the pre-sterilized hydrogel are critical in order to choose the most suitable machinery for the filling of the syringes.

A summary of the difficulties for scaling up the fabrication of injectable hydrogels and the suggested solutions are provided in Table 2.

### 4.2. In-House Example: Fabrication of an Injectable Hydrogel for Dermal Filling Applications

At i+Med laboratories, we have developed a biopolymer-based dermal filler from the lab scale (prototypes) up to pilot batches.

#### 4.2.1. Lab-Scale Fabrication

The fabrication of the lab scale prototypes was performed in the cleanroom so that the specification of visual inspection and microbiological burden were fulfilled. First assays were performed with the use of a glass flask. In this regard, a crosslinker containing reaction medium is poured in the glass flask, and the hydrogel-forming biopolymer, in powder form, is mechanically added and dispersed via spatula. Detailed work instructions of this step (dispersion time, spatula manipulation) are provided to the laboratory staff in order to avoid inter-operator influence in the resulting polymer matrix.

The next step involves the heating of the glass flask with the purpose of cross-linking the hydrogel. Monitorization of the temperature in the heating bath and inside the glass reactor is realized so that the chemical modification of the biopolymer proceeds homogeneously. Longer heating periods produce a higher cross-linking rate; therefore, the optimal heating time must be established.

Purification of the hydrogel that consists of the removal of the non-reacted crosslinker and of the low molecular weight side products is achieved by membrane dialysis in aqueous buffer. It is strongly recommended that dialysis membranes display a microbial burden as low as possible to avoid the degradation of the biopolymer during the dialysis process. For that, membranes stored in sodium azide or gamma-ray sterilized membranes can be purchased.

After purification of the hydrogel, the concentration of the biopolymer is adjusted via the addition of aqueous buffer. The mechanical mixing of the buffer and the hydrogel takes place with the aid of a spatula and a roller stirrer. Following, the hydrogel is loaded in the syringes semiautomatically. Finally, syringes are blister packed and sterilized.

At the lab-scale phase, we investigated the influence of the crosslinker concentration, temperature, reaction time, dialysis period, and mechanical dispersion in the rheological properties, syringeability, and injectability of the resulting polymer matrix.

#### 4.2.2. Development Batches

At this stage, the fabrication process is transferred from a glass flask to jacketed flask reactors located in the cleanroom. In this case, the dispersion of the biopolymer powder is achieved through stirring rods that possess mixing elements suitable for (very) high viscous solutions. The geometry of the mixing element, speed, and mixing time are critical for achieving a successful dispersion of the solid in the solution. Alternatively, the stirring rod can be replaced by a dispersing element, which allows for a faster dispersion of the biopolymer powder.

Heating of the flask reactor proceeds through the jacket, which provides a better control over the temperature than the use of heating baths. The temperature is monitored at several points of the reaction medium to assure that the crosslinking process takes place consistently. The transfer of the heating period from flasks to jacketed reactors is critical in order to get the same crosslinking rate with both systems.

Purification of the hydrogel occurs via a membrane dialysis as described previously. Mechanical mixing of the hydrogel with buffer for achieving the required concentration of the biopolymer is performed with the aid of a dispersing element. In this regard, the size parameters of the dispersing element determined the rheological parameters of the resulting product. Next, the hydrogel is packed in the syringes semiautomatically. After that, syringes are blister packed and sterilized.

The key parameters in the chemical process that determine the properties of the final product are the same as those in the lab-scale fabrication.

#### 4.2.3. Pilot Batches

Fabrication of our product at the pilot batch scale requires the use of industrial reactors of a volume depending on the final batch size, which is typically of 10 kg. Such reactors are located in environmentally controlled areas. The reactor set up includes a dispersing element as well as a heating system that allows the homogeneous heating of the biopolymer solution. The dispersion process is controlled through the speed, time, and size parameters of the dispersing element. Concerning this, the performance of the dispersing element must be able to reproduce the mixing obtained within the flask reactor.

Purification of the hydrogel is performed with a cartridge system that enables the simultaneous purification of several membranes. Alternatively, custom dialysis equipment can be designed for this process. Key parameters of the fabrication process are those indicated above. The scaling up to >10 kg implicates that the filling and emptying of the membrane must be performed with as little manipulation as possible. For that purpose, custom-made solutions are designed to meet the specific needs of the manufacturer. Bench-top and large production filling systems that allow the automatic filling of the syringes are commercially available.

### 4.3. Regulatory Aspects

Products from any sector containing or consisting of chemical substances and mixtures are subject to specific requirements under the Registration, Evaluation, Authorization and Restrictions of Chemicals (REACH) Regulation and the Classification, Labeling, and Packaging of substances and mixtures (CLP) Regulation. In any case, for medical devices such as injectable hydrogels specifically and additionally to REACH and CLP, the MDR (EU) 2017/745 is applied, as it regulates requirements for assessing and managing the risks (i.e., biological safety verification) and providing appropriate safety information to users.

Regarding the grade of the raw material, it is recommended to use pharmaceutical grade for the manufacture of medical devices in order to make sure the product is safe. A pharmaceutical grade compound is any active or inactive drug, biologic, or reagent for which a chemical purity standard has been established by a recognized pharmacopeia. These standards are used by manufacturers to help ensure that the products are of the appropriate chemical purity and quality, in the appropriate solution or compound, to ensure stability, safety, and efficacy.

Some of the tests that must be performed in order to guarantee the security of the substance are acute toxicity, skin irritation, eye irritation, respiratory, or skin sensitisation, cell mutagenity, embryotoxicity, carcinogenicity, and reproductive toxicity. If the substance is supposed to be injected, some more tests such as acute intravenous toxicity, or acute intraperitoneal toxicity must be performed, as well. All these tests are performed by the supplier of the raw material and provided to the manufacturer of the medical device upon request after a previously signed technical contract by both parties following the recommendation of ISO 10993-18. It is the responsibility of the manufacturer of the medical device to ensure the quality and safety of the final product, not of its components, but the better quality of the raw material, the better quality of the final medical device that should be obtained. For that purpose, the technical file of the raw material, MSDS, and other technical details are usually requested by the manufacturer to the supplier.

The safety evaluation of a medical device can be assessed by assessing the safety of the components, based on the literature of assays performed to similar products or by tests performed following ISO 10993.

According to the regulation, the necessary biocompatibility evaluation for each medical device is based on a categorization that depends on the category of the device (non-contacting, surface-contacting, external communication, or implant), the type of contact with the device (e.g., skin, tissue/bone, or blood), and the duration of contact (<24 h, >24 h to 30 days, >30 days). Injectable hydrogels are considered Class III medical devices and Rules 8 and 14 from EU 2017/745 are applied.

The process adapted for hydrogels to comply with the European regulation and to obtain the CE mark is as follows:

1. Determination of the regulation that applies, considering that hydrogels are active implantable medical devices.
2. Classify the medical device: Class I, IIa, IIb, or III. Hydrogels are Class III.
3. Implementation of the Quality Management System.
4. Elaboration of a technical file with all the available information, including physical, chemical, and technical characteristics and properties, and clinical data about the hydrogel in order to prove its compliance with the regulation.
5. Audit from a Notified Body of the QMS and technical file of the hydrogel. The clinical evaluation reports and the post-marketing surveillance activities must be also performed.
6. Obtaining the CE marking certificate (valid for three years) for the hydrogel and an ISO 13485 certificate (valid for one year) for the manufacturer facilities. In some countries, a manufacture license is also required.
7. Elaboration of a declaration of conformity declaring the compliance of the hydrogel to the corresponding regulation, where the CE marking certificate can be now attached.
8. Registration of hydrogels in those member states where their national regulation requests it. The process will be repeated once the CE mark/ISO 13485 certificate loses its validity.

**Figure 8:**
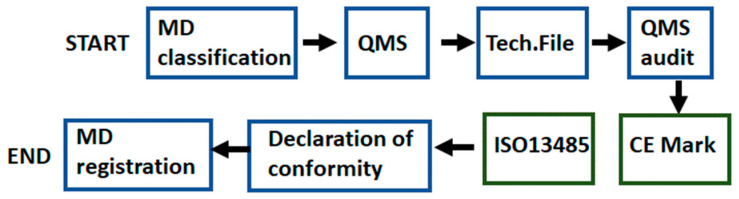
Schematic flow for medical devices to comply with the European regulation and to obtain the CE mark.

Figure 8 shows the schematic flow of the described process.

Considering these characteristics, the risk class for the hydrogel-based medical devices is the highest, and therefore, apart from the physicochemical characterization, the other test summarized in the table must be performed to comply with the defined requirements (Table 3). The table shows the different standards that apply to injectable medical devices based on hydrogels.

## 5. Conclusions

This review is intended to serve as a guide on the scaling up of injectable hydrogels for scientists who are non-initiated in industrial processes. In this regard, we discussed the requirements for the scaling up of injectable hydrogels from laboratory to the industrial level in terms of (i) synthesis techniques of injectable hydrogels, (ii) characterization techniques, (iii) safety and efficacy assays, (iv) process parameters (machinery, time, temperature, hydrogel purification, and filling of syringes) as well as (v) regulatory aspects. An example of an in-house-developed biopolymer-based dermal filler is presented showing the different steps that must be followed to get a reliable and safe product into the market. In opinion of the authors, the transfer of the process scale parameters from the laboratory to the industrial scale is the topic that requires the most effort in the near future.

## Figures and Tables

**Table 1:** The most relevant rheological parameters that describe the viscoelastic properties of injectable hydrogels.

Parameters	Formula	Definition and Characteristics
Viscosity (η)	Strain rate η = Stress	It is the flow characteristic of a gel and it is used to define gel thickness.
Elastic viscosity (η′)	$\eta^{\prime }=\frac{G^{\prime \prime }}{\omega }$	η′ is proportional to G″
Viscous viscosity (η”)	$\eta^{\prime \prime }=\frac{G^{\prime }}{\omega }$	η″ is proportional to G′
Complex viscosity (η*)	$\eta *=\eta^{\prime }+i\;\eta^{\prime \prime }$	It is the viscosity calculated from frequency sweep.
Elastic modulus (G′)	$G^{\prime }=\frac{Stress}{{Strain}^{\prime }}\;\cdot cos\delta$	It characterized the stored energy in a viscoelastic material. Higher G′ values correlates with a firmer gel.
Viscous modulus (G″)	$G^{\prime }=\frac{Stress}{{Strain}^{\prime }}\cdot sin\delta$	It measures the resistance to dynamic forces. Lower G″ values are less ticker gels and require less force to extrude through a needle.
Complex modulus (G*)	$G*=\sqrt{{\left(G^{\prime }\right)}^{2}+{\left(G^{\prime \prime }\right)}^{2}}$	It characterized the overall ability to resist de formation. Injectable gels possess G* equal to G′
Loss factor (tan *δ*)	$tan\delta =\frac{G^{\prime \prime }}{G^{\prime }}$	Loss factor measures the relative proportions of elastic to viscous modulus. Hydrogels with low loss factor (close to 0) are predominantly elastic.

**Table 2:** Difficulties and solution for scaling up the fabrication of injectable hydrogels.

Difficulties in Scaling up	Suggested Solutions
Rheological parameters	Rheological fluid behavior must be normalized to foresee the flow behaviors by comparison with the common standardization function (master curve) and to predict non-Newtonian fluid parameters based on Newtonian models
Mixing/dispersing of the hydrogel precursor	Adjust the propeller/dispersor configuration
Process time	Perform dimensional analysis based on Reynolds and Archimedes numbers
Process temperature	Sensors and software to control temperature depending chemical processes
Purification of the hydrogel	Comercial solution for scaling up purification steps
Filling of syringes with the hydrogel	Check the rheological properties of the pre-sterilized hydrogel in order to select the filling machinery

**Table 3:** Test to comply with regulation of medical devices.

ISO Standard	Characterization Test
ISO 10993-3:2018	Biological evaluation of medical devices. Part 3: Genotoxicity and carcinogenicity
ISO 10993-5:2018	Biological evaluation of medical devices. Part 5: Cytotoxicity
ISO 10993-6:2017	Biological evaluation of medical devices. Part 6: Tests for local effects after implantation
ISO 10993-10:2018	Biological evaluation of medical devices. Part 10: Tests for irritation and skin sensitization
ISO 10993-11:2018	Biological evaluation of medical devices. Part 11: Tests for systemic toxicity
UNE-EN ISO 11607-1:2017	Packaging for terminally sterilized medical devices. Part 1: Requirements for materials, sterile barrier systems, and packaging systems
UNE-EN ISO 11607-2:2017	Sterilization of medical devices—Microbiological methods. Part 2: Tests of sterility performed in the definition, validation, and maintenance of a sterilization process
UNE-EN ISO 11737-1:2018	Sterilization of health care products—Microbiological methods. Part 1: Determination of a population of microorganisms on product
UNE-EN ISO 11737-2:2010	Sterilization of medical devices—Microbiological methods. Part 2: Tests of sterility performed in the definition, validation, and maintenance of a sterilization process
UNE-EN ISO 13485:2018	Medical devices. Quality management systems. Requirements for regulatory purposes
UNE-EN ISO 14630:2013	Non-active surgical implants. General requirements
UNE EN ISO 14644-1: 2016	Cleanrooms and associated controlled environments. Part 1: Classification of air cleanliness by particle concentration
UNE-EN ISO 14644-4:2001	Cleanrooms and associated controlled environments. Part 4: Design, construction, and start-up
ISO 14971:2019	Medical devices. Application of risk management to medical devices.
UNE-EN ISO 15223-1:2017	Medical devices. Symbols to be used with medical device labels, labelling and information to be supplied. Part 1: General requirements
UNE-EN ISO 17665-1:2007	Sterilization of health care products—Moist heat. Part 1: Requirements for the development, validation, and routine control of a sterilization process for medical devices
UNE-EN 62366-1:2015	Medical devices. Part 1: Application of usability engineering to medical devices
UNE-ISO 2859-1:2012	Sampling procedures for inspection by attributes. Part 1: Sampling schemes indexed by acceptance quality limit (AQL) for lot-by-lot inspection
GMP. Annex 1.	Manufacture of Sterile Medicinal Products
ISO 16061:2015* *when needles are included	Instrumentation for use in association with non-active surgical implants. General requirements
UNE-ISO 2859-1:2012	Sampling procedures for inspection by attributes. Part 1: Sampling schemes indexed by acceptance quality limit (AQL) for lot-by-lot inspection

## Data Availability

No new data were created or analyzed in this study. Data sharing is not applicable to this article.
